# Pulmonary Endobronchial Hamartoma Presenting With Post-obstructive Pneumonia

**DOI:** 10.7759/cureus.60916

**Published:** 2024-05-23

**Authors:** Diane S Habib, Pushan Jani, Bihong Zhao, Elakiya Anjali Jayaraman, Ramesh Kesavan

**Affiliations:** 1 Internal Medicine, Hospital Corporation of America (HCA) Houston Healthcare Kingwood/University of Houston, Kingwood, USA; 2 Internal Medicine, University of Texas Health Science Center at Houston-McGovern Medical School, Houston, USA; 3 Combined Anatomic and Clinical Pathology, University of Texas Health Science Center at Houston-McGovern Medical School, Houston, USA; 4 Pulmonology, The Village School, Houston, USA; 5 Pulmonary and Critical Care, Hospital Corporation of America (HCA) Houston Healthcare Kingwood/University of Houston College of Medicine, Kingwood, USA

**Keywords:** endobronchial hamartoma, argon plasma photocoagulation, post-obstructive pneumonia, lung cryotherapy, pulmonary hamartoma

## Abstract

Pulmonary hamartomas (PH) are rare but are the most common benign tumors found in the lungs. They are slow-growing and are usually found incidentally on chest imaging during the sixth decade of life. Approximately 10% of pulmonary hamartomas are endobronchial. Rarely, pulmonary hamartomas can cause a spectrum of pulmonary symptoms depending on their size and location. We present a case of endobronchial hamartoma causing airway obstruction and recurrent post-obstructive pneumonia.

## Introduction

Endobronchial tumors are difficult to diagnose and rare. Most of the endobronchial lesions are malignant with around 11% of the lesions being benign [1]. Benign endobronchial lesions are slow-growing and present with vague symptoms such as chronic cough, wheezing, and chest pain [2]. Radiographic features are vague, making them a diagnostic challenge. Features include atelectasis, recurrent pneumonia, and bronchiectasis [3,4]. Various etiologies of benign endobronchial lesions such as anthracosis, tuberculosis, sarcoidosis, aspergillosis, hamartoma, lipoma, adenoma, and papilloma have been reported [1,2]. Approximately 22% of benign lesions present with airway obstruction, defined as greater than 50% occlusion [1]. Flexible bronchoscopy plays an important role in evaluating these endobronchial lesions and obtaining a pathologic diagnosis [5]. We present a case of endobronchial hamartoma causing airway obstruction and recurrent post-obstructive pneumonia.

## Case presentation

**Figure 1:**
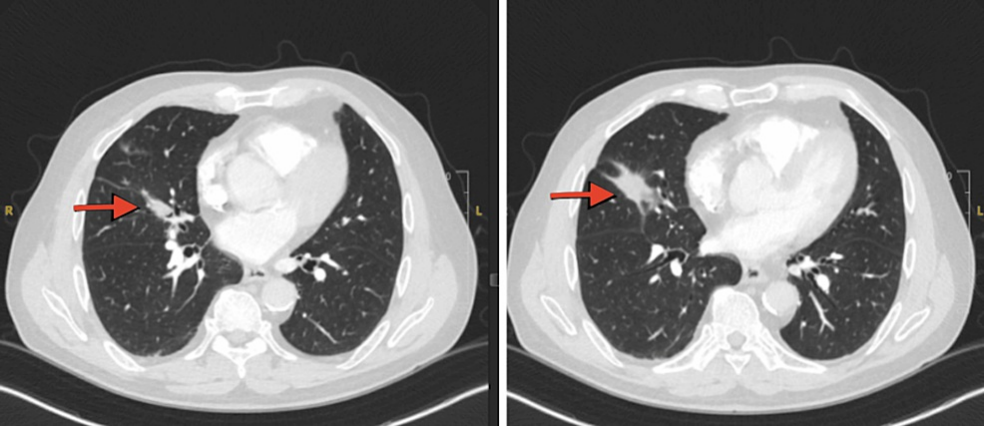
CT of the chest images showing evidence of right middle lobe endobronchial lesion (red arrowheads) resulting in focal post-obstructive collapse

**Figure 2:**
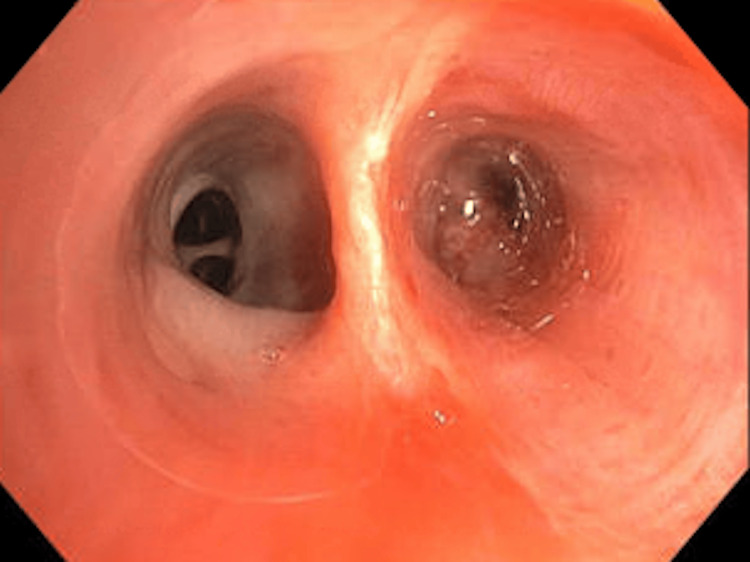
Endobronchial tumor in the right middle lobe lateral segmental bronchus

**Figure 3:**
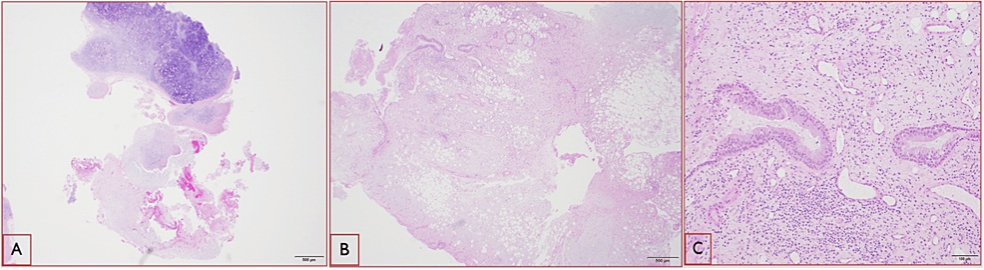
Pathology slides (A) Mature cartilaginous tissue with mild and focal calcification (hematoxylin and eosin {H&E}: ×20). (B) A mixture of different types of tissue and cells including adipose, fibrotic, and glandular tissues (H&E: ×20). (C) The higher power of the glandular tissue with respiratory-type epithelium (H&E: ×100)

A 71-year-old male with a past medical history of hypothyroidism, chronic obstructive lung disease, and tonsillar cancer, who had received chemotherapy and radiation five years ago, presented to the emergency room with two weeks of dry cough, dyspnea, and minimal blood-tinged sputum. The patient had previously been treated for community-acquired pneumonia as an outpatient, but his symptoms did not resolve despite treatment with a course of azithromycin, cefdinir, and prednisone prescribed one week prior to this visit. He had 20 pack-year smoking history in the past but quit five years ago. His oxygen saturation was 96% on room air, and his vital signs were otherwise stable. His lung examination was clear. His WBC count was 11000/microliter with 91% neutrophilia. A CT scan of the chest without contrast (Figure 1) revealed an endobronchial lesion in the right middle lobe (RML) segmental bronchus with a nodular appearance measuring 13 mm × 4 mm with punctate calcification. Findings were suspicious for an endobronchial lesion with post-obstructive lung collapse. During the bronchoscopy, a completely obstructing endobronchial mass was found proximally at the orifice of the lateral segment of the RML (Figure 2). Bronchoalveolar lavage (BAL) and endobronchial brushing were obtained from that segment, as well as endobronchial biopsies under fluoroscopy guidance. Pathology was consistent with a pulmonary hamartoma (PH) (Figure 3). The patient was successfully treated with debulking of the tumor using cryotherapy and argon plasma coagulation. Following debulking, post-obstructive purulent material was therapeutically aspirated, and the distal airway appeared patent.

## Discussion

Benign endobronchial tumors are rare and difficult to diagnose given their vague clinical and radiographic presentation [2]. PH are the most common benign tumor found in the lungs [6]. PH were originally described in 1904 by the German pathologist Eugen Albrecht [5]. They are composed of mature mesenchymal tissue commonly found in the lung that develops without preserving its architecture [7]. Given their slow growth, PH are usually found incidentally on chest imaging [5,8]. Endobronchial hamartomas are rare. Approximately 10% of PH are endobronchial, most of them being peripherally located [9]. Endobronchial hamartomas have a higher prevalence in male smokers in their fifth and sixth decade [10]. Rarely, PH can cause a spectrum of pulmonary symptoms depending on its size and location [11]. PH can present with a persistent cough, hemoptysis, pneumonia, pneumothorax, and even airway obstruction [11,12]. Endobronchial hamartomas can lead to recurrent pneumonia and bronchiectasis [13]. Endobronchial hamartomas can be misdiagnosed as asthma [14]. Patients with pulmonary hamartoma have an increased risk of lung cancer [5]. Intervention is necessary when the pulmonary hamartoma expands or becomes symptomatic as in our patient [15]. Treatment should be considered for asymptomatic patients since they can develop obstructive pneumonia and also potentially increased risk for malignancy [5,16,17]. Airway inspection with flexible bronchoscopy is essential to evaluate the airway and obtain a pathologic diagnosis [2]. Tumor debridement using techniques such as argon plasma coagulation, cryotherapy, and mechanical debulking using rigid or flexible bronchoscopy is being used and has shown to be well tolerated with no residual or recurrent disease on follow-up [18,19].

## Conclusions

Endobronchial lesions can cause vague symptoms and radiological findings. High clinical suspicion and careful radiological and airway evaluation with a bronchoscope are necessary to clinch the diagnosis. The majority of endobronchial lesions are malignant. Rarely, these lesions are benign. However, despite not being cancerous, these benign endobronchial lesions can cause various complications such as post-obstructive pneumonia, bronchiectasis, and a potential risk of malignancy. As discussed in our case, endobronchial hamartomas, even though rare, can lead to potential complications and warrant further management with advanced bronchoscopy techniques.
